# Social, Environmental, and Spiritual Quality of Life Among Women of Reproductive Age and Its Relationship With Nutritional Status: A Cross‑Sectional Study From the Center for Rural Health, All India Institute of Medical Sciences, Nutakki, India

**DOI:** 10.7759/cureus.109318

**Published:** 2026-05-20

**Authors:** Venkatashiva Reddy B, Sefin A P, Remya M John, Chandru R, Rajeev Aravindakshan

**Affiliations:** 1 Community and Family Medicine, All India Institute of Medical Sciences, Mangalagiri, Mangalagiri, IND

**Keywords:** nutritional, quality of life, rural health care, whoqol100, women of reproductive age

## Abstract

Introduction: Quality of life (QoL) is a key measure of well-being that encompasses environmental, social, and spiritual dimensions, which are especially important to understand among women of reproductive age in rural settings. The objective of the study was to estimate the mean environmental, social, and spiritual QoL among women of reproductive age and to study the associated factors.

Methods: This cross-sectional study was conducted in the outreach areas of a primary health center. Females aged 15-45 years living in the outreach areas for the last three months were included. The minimum sample size was calculated to be 110. Systematic random sampling was done. A pretested questionnaire based on the World Health Organization Quality of Life - 100 items (WHOQOL-100) was used to collect data.

Result: The mean (SD) age of 110 participants was 35.6 (7.6) years. The participants obtained a mean (SD) score of 11.6 (1.4), 12.5 (1.3), and 11.5 (3.6) for the social, environmental, and spiritual domains of QoL, respectively. In binomial regression, participants whose husbands had gone to colleges, did formal jobs, who lived in pucca houses, and owned agricultural lands had statistically significantly higher odds of having higher QoL in the social domain. Participants owning agricultural land had higher odds of having higher QoL in the environment domain. The presence of livestock was, however, found to be associated with the spiritual domain of QoL after adjusting for housing type. Even though 78 (70.91%) of women had anemia, there was no significant association between the presence of anemia and the social, environmental, and spiritual domains of QoL.

Conclusion: This study shows that women's QoL depends on household conditions such as the presence of livestock. Single-focus programs may fall short; integrated approaches addressing these factors are needed for real improvement of the QoL of women.

## Introduction

Quality of life (QoL) is defined as "individuals' perception of their position in life in the context of the culture and value systems in which they live and in relation to their goals, expectations, standards, and concerns. This is a very broad concept, and one that can be influenced in a complex way by the physical health of the subject, his or her psychological state and level of independence, social relations, and relationship with the essential elements of his or her environment" [1]. QoL provides a comprehensive understanding of how health conditions influence daily functioning, life satisfaction, and overall well-being. It is founded on several objective factors, such as the quality of the environment and living conditions, and subjective factors measured in terms of satisfaction. Health status as an essential component of QoL is referred to as health-related QoL (HRQOL) [2-4].

Women of reproductive age play a pivotal role in family and community health. However, hormonal, physical, and psychosocial pathways influence women's QoL. Menstruation, premenstrual dysphoric disorder, pregnancy, infertility, and miscarriage elevate anxiety/depression risk. It impairs social QoL amid sleep loss and body changes and erodes spiritual well-being and self-esteem via stigma and treatment stress. They are also affected by nutritional deficiencies, especially anaemia [5-7].

Anaemia remains highly prevalent among women in India despite ongoing national control efforts. However, while clinical management is well established, its broader impact on non-physical domains of QoL remains underexplored [8]. Beyond physiological manifestations, such as fatigue, reduced physical endurance, and impaired immunity, anaemia can limit women's ability to perform daily activities, engage in employment, and maintain caregiving roles, thereby affecting the physical domain of QoL [9]. Chronic ill-health and reduced functional capacity may also influence psychological well-being, contributing to diminished self-esteem, emotional distress, and reduced coping capacity [10]. In the sociocultural context of India, where women often shoulder multiple domestic and social responsibilities, compromised health can strain interpersonal relationships and reduce participation in family and community life, thereby affecting the social domain of QoL [11,12]. Environmental factors such as access to health services, financial resources, transportation, and living conditions further shape both anaemia risk and QoL [13]. Previous studies among women of reproductive age in India have largely focused on physical and psychological domains of QoL, often in relation to anaemia, pregnancy, or chronic illness. However, limited evidence is available from rural settings, particularly from South India and Andhra Pradesh, examining the social, environmental, and spiritual domains of QoL. This highlights an important contextual evidence gap. The objective of this study was to estimate the mean environmental, social, and spiritual QoL among women of reproductive age in the outreach area of a primary healthcare centre (PHC), to identify factors associated with these domains, and to assess their relationship with anaemia.

## Materials and methods

Study design

This study was conducted as a cross-sectional study in the outreach area of the Center for Rural Health, All India Institute of Medical Sciences (AIIMS), PHC Nutakki in Guntur, South India. Guntur is one of the 28 districts of Andhra Pradesh, India. It has two administrative revenue divisions, comprising 18 mandals, and caters to approximately 54 lakh population. The outreach program of the PHC serves nine nearby villages, covering about 18,000 people [14].

Study participants

The inclusion criteria for the study were females aged 15-45 years (women of reproductive age) living in the outreach areas for at least the past three months, who were married, were homemakers, and had an education level of intermediate or higher. Women with intellectual disability who did not give consent and were sick at the time of the interview were excluded from the study.

Sample size and sampling

The sample size was calculated based on the standard deviation of the environmental QoL domain from a multi-centric World Health Organization Quality of Life - 100 items (WHOQOL-100) study conducted across 35 countries, including India (mean = 63.28, SD = 14.95) [15]. This domain was chosen because it showed higher variability compared to the social relations and spiritual domains, providing a more conservative estimate to ensure adequate power for all domains. The social relations domain had a lower standard deviation, indicating less variability, while the spiritual domain is a single-facet domain and was therefore not considered suitable for sample size calculation. Using the environmental domain allowed for a robust estimation of the required sample size. With a precision of 3 and a 95% confidence interval, the minimum sample size was calculated to be 96. After accounting for a 15% non-response rate, the final sample size was set at 110. Systematic random sampling was done to identify the participants. The number of households in the population was known. The sampling interval was calculated. A house was selected at random, and the sampling interval was used to identify the subsequent houses till the sample size was achieved. The data for the study were collected from October 2025 to January 2026.

Study tool

A pre-tested semi-structured questionnaire was used to collect the data. The questionnaire included questions on socio-demographic characteristics, such as age and housing standards, as well as questions from the WHOQOL-100 on social, environmental, and spiritual QoL [4]. WHOQOL-100 has 25 facets of QoL clustered and scored within one of six domains reflecting its conceptual structure: physical, psychological, independence, social, environmental, and spiritual QoL [16]. This study specifically examined the social, environmental, and spiritual domains of QoL, as these are more sensitive to sociocultural, livelihood, and contextual influences in rural settings, particularly among women of reproductive age. While physical and psychological domains have been widely studied in relation to anaemia, there is limited evidence on the impact of anaemia and socio-economic factors on these broader dimensions.

Hemoglobin Measurement

Hemoglobin estimation was performed using the Hb 301 hemoglobinometer (HemoCue AB, Ängelholm, Sweden) with on-spot capillary blood sampling. Universal precautions were followed throughout the procedure. After ensuring that the participant's hands were warm and relaxed, the middle or ring finger was selected for sampling. The fingertip was cleaned with disinfectant and allowed to dry completely. Using a sterile lancet, a finger-prick sample was obtained from the lateral side of the fingertip. The initial two to three drops of blood were wiped away, and the subsequent drop was collected into a microcuvette without air bubbles. The filled microcuvette was inserted into the Hb 301 device, and the hemoglobin value was displayed within three seconds. All procedures were carried out using appropriate personal protective measures, and biomedical waste was disposed of according to the Biomedical Waste Management Rules.

Anaemia was defined as per the cut-off of Hb concentration given by the World Health Organization in 2024 [17]. Anaemia severity was classified as mild (Hb: 11.0-11.9 g/dL), moderate (Hb: 8.0-10.9 g/dL), and severe (Hb: <8.0 g/dL) for non-pregnant women. The study participants were interviewed according to the pre-tested questionnaire, followed by anthropometric measurement and blood sampling. Universal safety precautions and biomedical waste management rules were strictly followed.

Environment, social, and spiritual QoL were the dependent variables, while demographic factors, socio-economic factors, and anaemia were the independent variables. Environment, social, and spiritual QoL was calculated using WHOQOL-100 with questions referencing the last two weeks. For logistic regression analysis, QoL domain scores were dichotomized into higher and lower categories based on the median value of each domain score. 

Statistical analysis

Data were entered in Microsoft Excel 2010 (Microsoft® Corp., Redmond, WA). After cleaning the data, it was imported into Statistical Product and Service Solutions (SPSS, version 28.0; IBM SPSS Statistics for Windows, Armonk, NY). The continuous variables were expressed as mean (SD) or median (IQR) based on the distribution of data. The categorical variables were expressed using proportions (%). An appropriate test of significance was used based on the distribution of data. Binary Logistic regression was done to calculate the unadjusted odds ratio (uOR). We performed a multinomial logistic regression using the backward stepwise method. Variables with a p-value < 0.20 in binary logistic regression analysis were included in the multinomial logistic regression model to avoid excluding potential confounders. Variables were then removed step-by-step if their removal did not worsen the model fit. The final model's goodness-of-fit was confirmed using the log likelihood ratio. Statistical significance in the final model was set at p < 0.05. 

Ethical consideration

The participants were provided with a participant information sheet that explained the objectives and procedure of the study and the rights of the participants. After obtaining informed written consent, the participant was included in the study. The study was approved by the Institute Ethics Committee for AIIMS Mangalagiri.

## Results

The mean (SD) age of 110 participants was 35.6 (7.6) years, and 79 (71.2%) were aged more than 30 years. About 104 (94.6%) participants had their own house, and 97 (88.2%) resided in pucca houses, 10 (9.1%) in semi-pucca houses, and 3 (2.7%) in kutcha houses. All households had their own flush toilet facility (110) and used liquefied petroleum gas for cooking (110). About 107 (97.3%) had a separate kitchen at home. The participants had a 4.0 (1.1) mean (SD) number of people in their family and a median (IQR) of two (one) siblings. The mean (SD) number of rooms per household was 2.2 (0.5). About 86 (78.2%) participants obtained drinking water from wells, whereas 24 (21.8%) relied on public taps or other sources. Agricultural land ownership was reported by 27 (24.6%) participants, and 10 (9.1%) reported owning livestock, as described in Table 1.

**Table 1:** Distribution of participants according to socio-demographic characteristics (n=110)

Variable	Frequency (%)
Age (in years)	
Less than 30 years	31 (28.2)
More than 30 years	79 (71.8)
Education of Husband	
Illiterate	13 (11.8)
Primary school	17 (15.5)
Middle school	20 (18.2)
High school	41 (37.3)
Graduate and above	14 (12.7)
Occupation of Husband	
Daily wage labourer	54 (49.1)
Farmer	25 (22.7)
Private job	15 (13.6)
Small business	9 (8.2)
Government job	3 (2.7)
No employment	2 (1.8)
Type of House	
Pucca	97 (88.2)
Semi-pucca	10 (9.1)
Kutcha	3 (2.7)
House Ownership	
Own	104 (94.6)
Rented	6 (5.4)
Agricultural Land Ownership	
Yes	27 (24.6)
No	83 (75.5)
Livestock Ownership	
Yes	10 (9.1)
No	100 (90.9)

**Figure 1:**
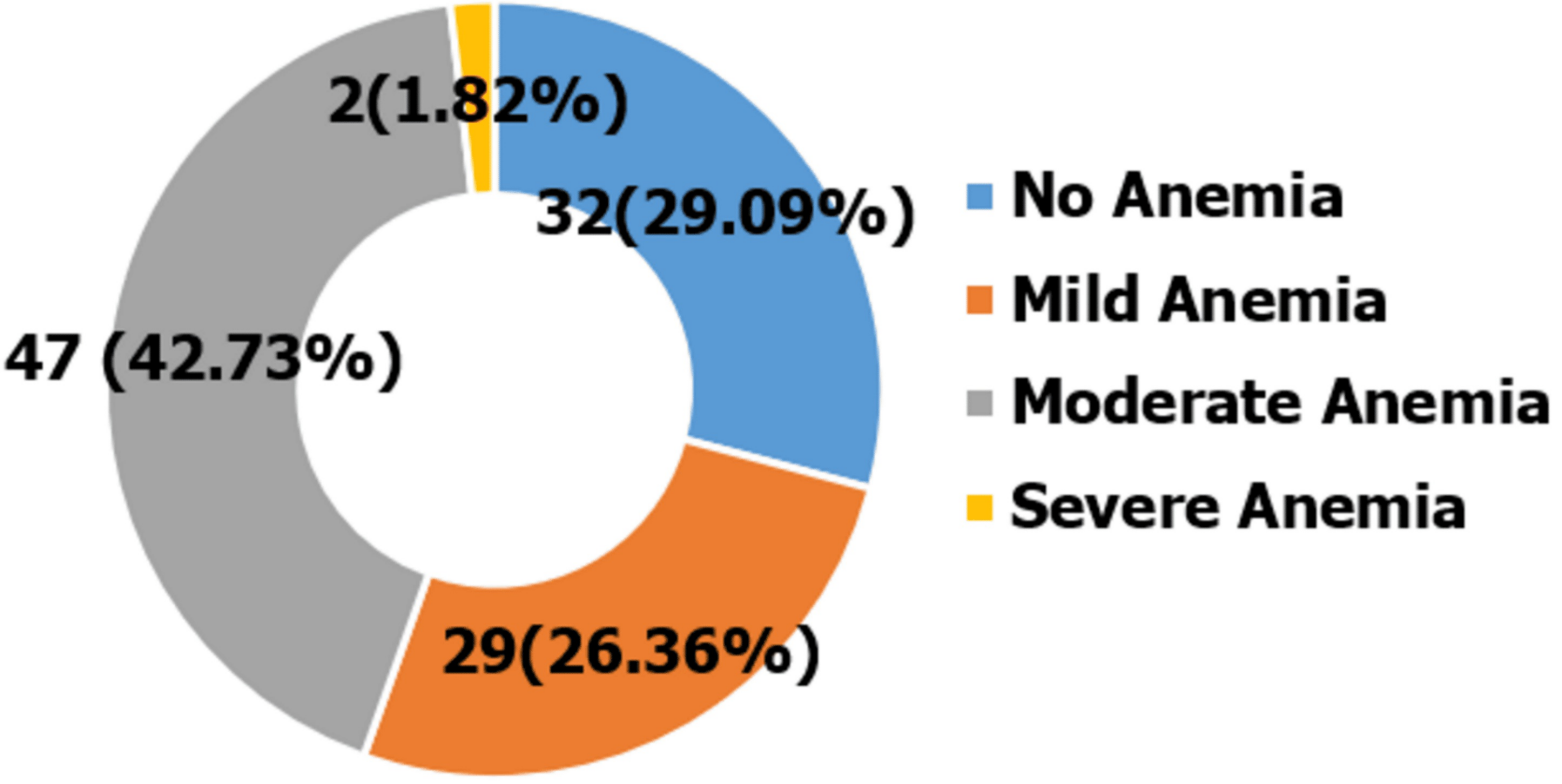
Distribution of participants based on severity of anaemia (n=110)

Out of 110 participants, only 32 (29.09%) had Hb more than 12 g/dL. Among the 78 participants with Hb less than 12 g/dL, 29 (26.6%) had more than 11 g/dL Hb (mild anaemia), 47 (43.1%) had between 8 and 11 g/dL (moderate anaemia), and 2 (1.8%) had less than 8 g/dL (severe anaemia), as illustrated in Figure 1.

**Figure 2:**
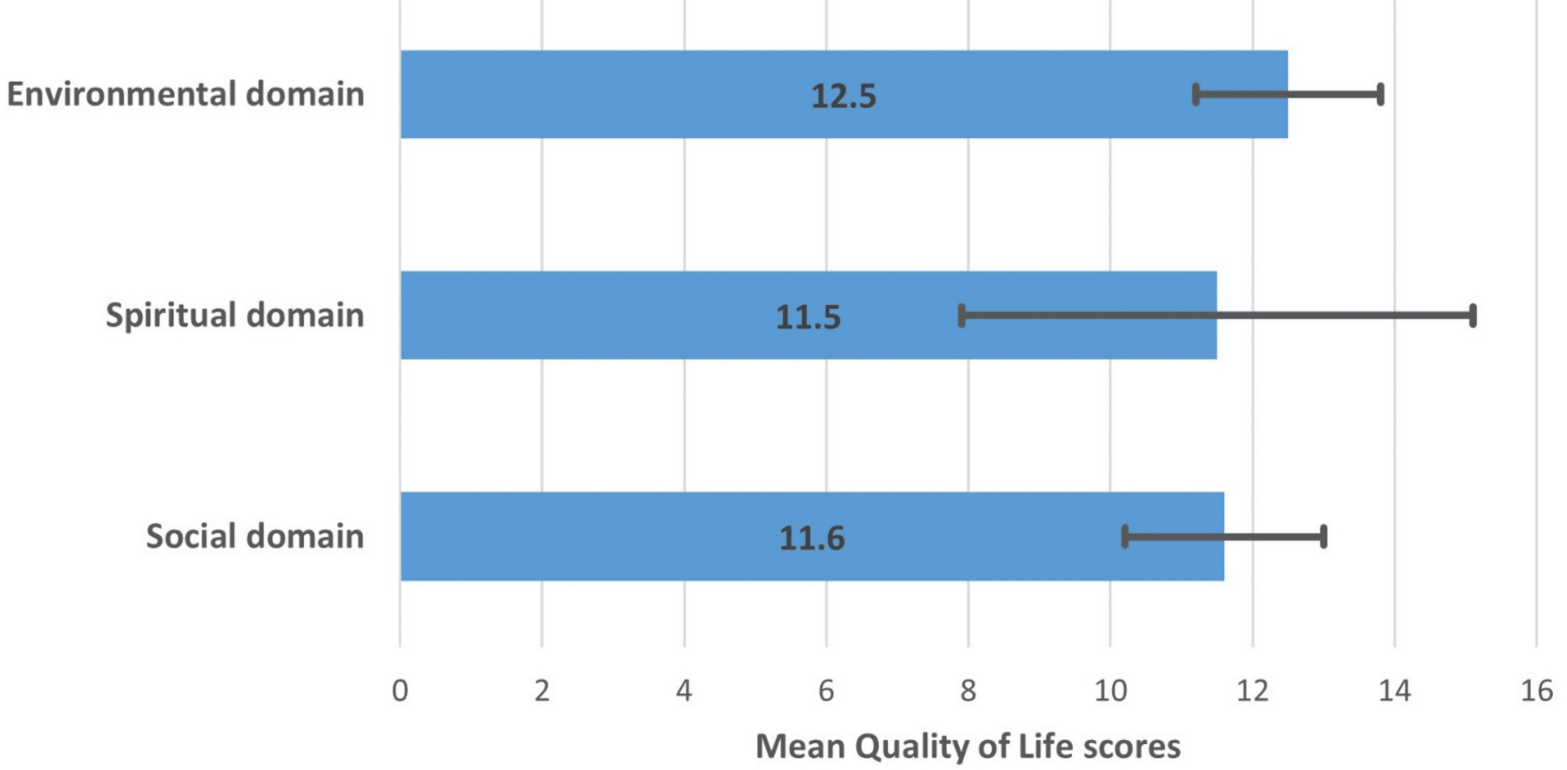
Distribution of WHO quality of life domain scores (mean ± SD)

Among the 110 participants, the mean (SD) QoL score for the social domain was 11.6 (1.4). The maximum score obtained was 14, and the minimum score was 6. The mean (SD) QoL score for the environment domain was 12.5 (1.3). The maximum score was 16, and the minimum score was 8. The mean (SD) QoL score for the spiritual domain was 11.5 (3.6), as shown in Figure 2. The participants scored a maximum of 20 and a minimum of 5 for the spiritual domain. The social QoL had a median score of 11.89, with an interquartile range (IQR) of 1.22. The environmental QoL had a median of 12.71, with an IQR of 1.29. The spiritual QoL had a median score of 10.00, with an IQR of 5.00.

In the binary logistic regression done (Table 2), participants whose husband had gone to colleges (uOR (95%CI): 7.9 (1.56-39.8), p = 0.012), whose husband did formal jobs (uOR (95%CI): 2.9 (1.2-7.6), p = 0.022), who lived in pucca houses (uOR (95%CI): 6.9 (1.4-32.8), p = 0.015), and who owned agricultural lands (uOR (95%CI): 2.9 (1.2-7.5), p = 0.023) had a statistically significant higher odds of having higher QoL in social domain. Age, anaemia status, house ownership, and livestock ownership were not significantly associated with the social domain in univariate analysis. Participants owning agricultural land had higher odds (uOR (95%CI): 3.4 (1.3-8.9), p = 0.014) of having higher QoL in the environment domain compared to those who did not own agricultural land, with a p value of 0.053. Anaemia was not significantly associated with any of the QoL domains. In univariate analysis, anaemia showed no significant association with social (uOR: 0.8; 95% CI: 0.3-1.8; p = 0.565), environmental (uOR: 1.1; 95% CI: 0.4-2.4; p = 0.903), or spiritual domains (uOR: 0.9; 95% CI: 0.4-2.1; p = 0.786).

**Table 2:** Binomial logistic regression of socio-demographic factors and anaemia status with social, environmental, and spiritual domains of quality of life uOR: unadjusted odds ratio

Variable	Social domain		Environmental domain		Spiritual domain	
	uOR (95% CI)	P value	uOR (95% CI)	P value	uOR (95% CI)	P value
Age (in years)						
<30	Reference	-	Reference	-	Reference	-
≥30	0.5 (0.2-1.1)	0.077	0.6 (0.2-1.3)	0.194	0.6 (0.3-1.5)	0.292
Anaemia						
No	Reference	-	Reference	-		
Yes	0.8 (0.3-1.8)	0.565	1.1 (0.4-2.4)	0.903	0.9 (0.4-2.1)	0.786
Education Status of Husband						
Illiterate	Reference	-	Reference	-		
Schooling	2.1 (0.6-7.3)	0.252	0.8 (0.2-2.5)	0.666	1.2 (0.4-4.0)	0.757
Higher education	7.9 (1.56-39.8)	0.012	1.7 (0.4-7.4)	0.471	1.6 (0.4-6.8)	0.525
Occupation of Husband						
Unemployed/dead	1.2 (0.2-9.4)	0.823	0.4 (0.0-3.7)	0.399	4.2 (0.4-41.9)	0.224
Informal employment	Reference	-	Reference	-	Reference	-
Formal employment	2.9 (1.2-7.6)	0.022	2.2 (0.9-5.6)	0.088	1.1 (0.5-2.7)	0.808
Type of House						
Non-pucca	Reference	-	Reference	-		
Pucca	6.9 (1.4-32.8)	0.015	1.9 (0.5-7.2)	0.299	2.9 (0.7-11.1)	0.124
Own House Present						
No	Reference	-	Reference	-		
Yes	0.5 (0.1-2.9)	0.435	0.5 (0.1-2.9)	0.449	1.6 (0.3-9.0)	0.603
Agricultural Land						
No	Reference	-	Reference	-		
Present	2.9 (1.2-7.5)	0.023	3.4 (1.3-8.9)	0.014	0.6 (0.2-1.4)	0.217
Has Livestock						
No	Reference	-	Reference	-		
Yes	2.4 (0.6-9.9)	0.217	3.6 (0.7-18.4)	0.118	0.1 (0.2-1.0)	0.053

However, in the multiple variable regression done, age of women, husbands' education, husbands' employment status, housing type, and presence of agricultural land at the house were not found statistically associated with social domains of QoL. Employment status of husband, age of women, and presence of agricultural land and livestock were also not statistically associated with the environment domain of QoL. The presence of livestock was, however, found statistically associated (aOR (95% CI): 0.12 (0.01-0.99), p = 0.048) with the spiritual domain of QoL after adjusting for housing type (Table 3).

**Table 3:** Multinominal logistic regression of socio-demographic factors and anaemia status with social, environmental, and spiritual domains of quality of life *Statistically significant; AOR: Adjusted odds ratio

Variables	Social domain AOR (95% CI)	P value	Environmental domain AOR (95% CI)	P value	Spiritual domain AOR (95% CI)	P value
Age (in years)						
<30	Reference		Reference			
≥30	0.62 (0.24-1.63)	0.332	0.65 (0.25-1.65)	0.363	-	-
Education Status of Husband						
Illiterate	Reference					
Schooling	1.30 (0.30-5.65)	0.724	-	-	-	-
Higher education	4.56 (0.59-35.09)	0.145	-	-	-	-
Occupation of Husband						
Unemployed/dead	Reference		Reference			
Informal employment	1.42 (0.10-19.20)	0.794	2.85 (0.27-29.95)	0.382	-	-
Formal employment	0.79 (0.05-11.72)	0.864	5.79 (0.-73.78)	0.176	-	-
Type of House						
Non-pucca	Reference				Reference	
Pucca	4.37 (0.83-23.07)	0.082	-	-	3.06 (0.78-11.97)	0.109
Agricultural Land						
No	Reference		Reference			
Present	2.57 (0.93-7.06)	0.068	2.45 (0.81-7.40)	0.113	-	-
Has Livestock						
No			Reference		Reference	
Yes	-	-	2.54 (0.43-15.13)	0.305	0.12 (0.01-0.99)	0.048*

## Discussion

This study assessed QoL across social, environmental, and spiritual domains among women of reproductive age and examined their association with selected socio-demographic and health-related factors. Socio-economic status, specifically the husband's level of education, employment, housing quality, and ownership of agricultural property, was associated with the social domain of QoL.

Participants were more likely to have a higher QoL in the social domain if their husbands had a college degree. Education enhances social engagement, communication skills, information availability, and household decision-making authority. The husband's occupation was another factor. Compared to those who did hard labour, participants whose husbands held desk jobs or professions reported a higher social QoL. This could be due to increased work-life balance, less physical strain, and better income stability, and hence a favourable impact on social connections and family ties. Higher social QoL scores were associated with people living in a pucca house and owning agricultural lands. Self-esteem, social engagement, and perceived respect in the community can all be impacted by housing quality, which frequently indicates long-term financial stability and social standing. Owning agricultural land is also a sign of social standing and economic stability in rural areas. Other QoL studies done among women of reproductive age group also report similar findings [1,15,18,19].

In the environmental domain, agricultural land ownership was found to be associated with QoL, though not statistically significant. Ownership of land may contribute to better environmental QoL by ensuring financial security, food availability, and a sense of control over one's living and working environment. Studies from India and other countries have similarly highlighted the role of livelihood-related assets in shaping environmental satisfaction and perceived safety [15,20]. Other factors, such as age, education, occupation, and housing type, did not show significant associations with the environmental domain in this study. However, other studies have reported an increase in environmental QoL with age. Achievements in career and economy as age increases might have resulted in better QoL at higher ages [21].

In the multivariable regression analysis, no factors showed a significant association with QoL in the environmental and social domains. This difference from the binomial analysis may be due to collinearity among closely related socio-economic variables, such as housing type, land ownership, education, and occupation. Livestock ownership was significantly associated with lower spiritual QoL in the multivariable analysis. This finding may reflect increased daily responsibilities and workload among women involved in livestock-related activities [22].

Although a large proportion of participants were anaemic, anaemia status was not significantly associated with any QoL domains. The lack of association between anaemia and QoL in this study warrants further consideration. The majority of participants had mild-to-moderate anaemia, which may not produce symptoms severe enough to significantly influence perceived QoL. In rural settings, symptoms such as fatigue and reduced endurance are often normalised as part of daily life, leading to under-recognition of their impact. Additionally, this study assessed social, environmental, and spiritual domains of QoL, whereas anaemia is more likely to affect the physical and, to some extent, psychological domains. Therefore, the absence of the physical domain in the present analysis may have limited the ability to detect an association. Furthermore, while WHOQOL-100 is a comprehensive tool, it may not be sufficiently sensitive to capture subtle functional impairments associated with mild anaemia. Similar observations have been made in rural Indian populations where chronic nutritional deficiencies are common and often under-recognised [23-25].

The findings suggest that improving QoL for women in rural settings should focus on enhancing education, employment opportunities, housing, and livelihood security. Addressing these socio-economic factors may positively influence social and environmental QoL domains.

Strengths and limitations

The study used a standardised QoL instrument and included multiple domains, allowing a comprehensive assessment. However, the cross-sectional design limits causal inference. The use of the odds ratio as a measure of association might have overestimated the strength of the association. Social desirability bias cannot be excluded. One limitation of the study is the use of systematic random sampling, which may introduce bias if there is an underlying pattern in the population list that coincides with the sampling interval. This could affect the representativeness of the sample and limit the generalizability of the findings. Additionally, systematic sampling assumes a random starting point and consistent population structure, which may not always hold in community-based settings.

## Conclusions

This study suggests that the social and environmental QoL among women of reproductive age is influenced by the broader socio-economic context of the household, rather than by individual factors acting alone. The absence of independent associations in multivariable analysis indicates that these factors are closely interconnected and that programme strategies focusing on single determinants may have limited impact unless underlying household-level disadvantage is addressed more comprehensively. The inverse association between livestock ownership and spiritual QoL draws attention to a possible unintended effect of livelihood-related interventions. While such assets may improve economic security, they may also increase daily responsibilities for women, potentially affecting their sense of well-being. This highlights the importance of designing programmes that are sensitive to women's workload, incorporating measures such as labour-sharing, time-saving strategies, and supportive systems within households. Despite the high prevalence of anaemia in this population, no association was observed with any domain of QoL. This finding is important, as it suggests that routine anaemia control programmes may not necessarily translate into perceived improvements in well-being. It also highlights the need for programme evaluations to include functional outcomes and patient-reported measures that better reflect lived experiences. Overall, these findings suggest that efforts to improve QoL should move beyond isolated interventions and adopt a more integrated approach. Addressing socio-economic conditions alongside women's daily workload and lived realities may be more effective in producing meaningful change. Future research should evaluate such multidimensional approaches using longitudinal and mixed-method designs to better understand how different factors interact to shape QoL.
